# Study of the formation processes of gold droplet arrays on Si substrates by high temperature anneals

**DOI:** 10.1186/1556-276X-6-151

**Published:** 2011-02-16

**Authors:** Alla Klimovskaya, Andrey Sarikov, Yury Pedchenko, Andrey Voroshchenko, Oksana Lytvyn, Alexandr Stadnik

**Affiliations:** 1 1V. Lashkarev Institute of Semiconductor Physics, National Academy of Sciences of Ukraine, 41 Nauki Avenue, 03028 Kiev, Ukraine

## Abstract

In this study, the peculiarities of the transformations of gold films deposited on the Si wafer surfaces as a result of high temperature anneals are investigated experimentally depending on the conditions of wafer surface preparation and the annealing regimes. The morphology and the distribution functions of the crystallites of gold films as well as the gold droplets formed as a result of anneals are studied as functions of annealing temperature, type of annealing (rapid thermal or rapid furnace annealing), and the state of the surface of Si wafers. The results obtained can be used for the controlled preparation of the arrays of catalytic gold droplets for subsequent growth of Si wire-like crystals.

## Introduction

Semiconductor Si wire-like crystals grown on Si substrates using the catalytic gold droplets have been studied since 1960 as prospective structures for the development of micro- and nano-electronic devices [1]. In the typical schema of the experiment, the gold droplets are first formed on the Si substrates. The growth process proceeds with the inlet flow of reactive gas that consists of Si-containing molecules (monosilane is a typical example) into the growth chamber [2,3]. The preferential decomposition of reactive gas molecules and the silicon incorporation in the positions of droplets take place, which cause the growth of elongated wire-like crystals, diameters of which are determined by the diameters of droplets. The droplet caps remain on the top of wires to enable the continuous catalytic process of the decomposition of Si-containing reactive species from the gas phase, the preferential Si incorporation into the droplets, transportation within them and/or on the cap surface, and incorporation in the wires growing at their interfaces with the droplets.

The initial system for the Si wire growth before the inlet flow of active gas mixture is the catalytic gold droplet array on the surface of Si substrate. The ensemble of catalytically active droplets can be formed by different techniques such as patterned metal deposition [4,5], self-aggregation in the droplets during metal deposition [6], or temperature-stimulated disjoining of a solid metal film deposited onto Si substrate [3,7]. The metal catalyst can undergo additional argon plasma etching to assist the disjoining of metal film and the formation of catalytic islands [8,9]. The regime of thermal treatment before the wire-like crystal growth determines the evolution kinetics of droplet ensemble and, hence, the properties of the subsequent process of Si wire growth.

This article presents the results of an experimental investigation of the peculiarities of the formation of the arrays of gold islands in the course of high temperature anneals of Si wafers with gold films deposited on their surfaces depending on the conditions of wafer surface preparation and annealing regimes.

## Experimental

The experiments were carried out on 500-μm-thick, (111)-oriented, boron-doped Cz-Si wafers with resistivity of 10 Ω cm obtained from two sources, namely, Wacker-Chemitronic GmbH, Germany, and Silicon Ltd., Ukraine. Before the deposition of gold films, the surfaces of Si wafers were made to undergo one of the two treatments, namely, (i) degreasing in acetone vapour without removal of native oxide or (ii) growing a uniform stoichiometric silicon oxide film by thermal oxidation in vacuum (the native oxide was removed in 5% HF with subsequent 5-10 min. rinsing in deionised water before this procedure and the wafers were subsequently annealed in hydrogen atmosphere during 40 min at 450°C). The thicknesses of oxide films were monitored ellipsometrically.

Thin films of gold (3 and 5 nm thicknesses) were deposited on Si wafer surfaces by vacuum sputtering at a pressure below 2 × 10^-6 ^Torr. Prepared structures were annealed to initiate the formation of the arrays of golden islands on the surface of Si substrates. Two methods of annealing were applied, namely, rapid thermal anneals (RTAs) and quick furnace anneals.

The RTA treatments were realised by the illumination of structures under investigation by linear halogen lamps whose emission maximum wavelength was 1 μm. The treatment temperatures were in the range of 300-1100°С. Linear halogen lamps were arranged in two parallel rows on both sides of samples to enable faster temperature growth (up to 70°С/s) and reduced thermal gradients that produce thermal-mechanical stresses. To avoid uncontrolled oxidation of the sample during the RTA treatments, the RTA chamber was refilled with argon at atmospheric pressure before each treatment cycle.

Short-time furnace anneals were carried out in Ar gas atmosphere by rapid insertion of samples in the heated zone in the central part of furnace reactor. The annealing temperature was in the range of 900-1050°C, the duration in the range of 10-20 s, and the pressure of Ar in the gas chamber corresponded to 1 atmosphere.

The control over the thicknesses of deposited gold films, their morphology as well as monitoring of changes produced by annealing were done by scanning atomic force microscopy (AFM) (NanoScope IIIa).

## Results and discussion

**Figure 1:**
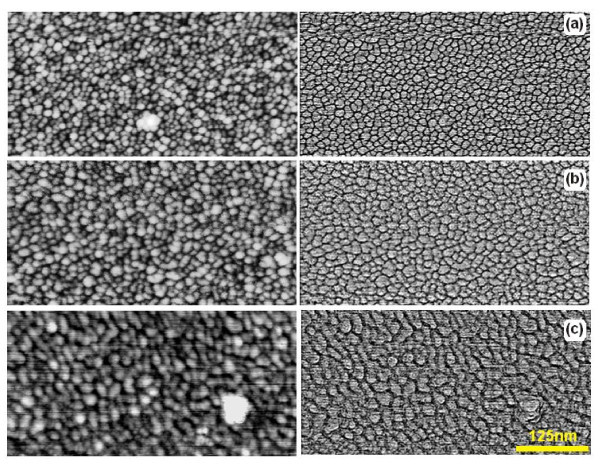
**AFM images of 3-nm-thick gold films deposited on Silicon Ltd. Si substrates with different surface oxide layer states**: **(a) **initial substrate with 2.4-nm-thick natural oxide layer; **(b) **substrate with an oxide layer modified by RTA (650°C, 15 s, oxide thickness after RTA is 1.7 nm); and **(c) **substrate after RTA (950°C, 15 s) with a 3.3-nm-thick modified oxide layer. The maps of heights are shown on the left-hand side; the same maps with distinguished grain boundaries are shown on the right-hand side. Numerical grain parameters are shown in Figure 2.

**Figure 2:**
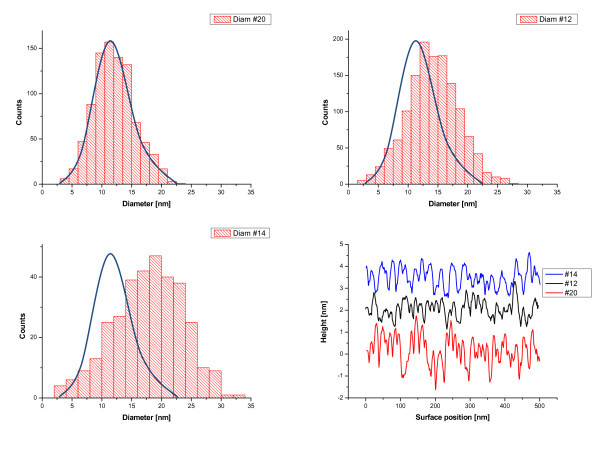
**Histograms of the distributions of the characteristics of gold films presented in Figure 1**: grain diameters **(a-c) **and the surface profiles **(d)**. For a good layout, the distribution **(a) **is superimposed on distributions **(b,c)**.

The development of the structure of gold films deposited onto the Si substrates takes place already during the stage of film deposition. Gold films acquire different nanocrystalline structures depending on the state of the oxide on the Si wafer. The AFM images of gold films deposited on the Si surfaces with different states of oxide coverage are shown in Figure 1. The size distributions of the crystallites formed are shown in Figure 2. As can be seen from these figures, the gold films formed crystallites with a typical size of 12 nm on substrates with native oxide after cleaning in neutral solution (Figure 2a). The nanocrystal sizes increased with the increase of annealing temperature for RTA-treated Si wafers (to 15 and 18 nm for RTA at 650 and 950°C, respectively, see Figure 2b,c). Such behaviour was mainly caused by the increase in surface homogeneity of native silicon oxide coverage that is supported by the surface profiles shown in Figure 2d. The gold films grown on initial Si surfaces had the most developed surfaces (RMS = 0.6 nm). RTA treatments led to the decrease of this value down to 0.4 nm. The histograms in Figure 2a,b,c demonstrate additionally the increase in the mean diameter of gold crystallites as a result of the increase of RTA temperature and the decrease of gold film roughness. It follows therefore that through modifying the oxide coverage on the surface of Si substrates, one can control the deposited gold film structure and subsequently, the process of the formation of catalytically active nanoislands for the growth of Si wire-like crystals.

**Figure 3:**
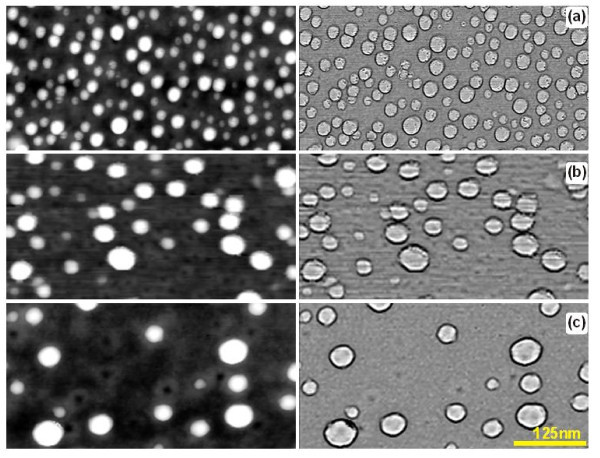
**AFM images of the surfaces of 3-nm-thick gold film deposited onto Silicon Ltd. Si substrates with the natural oxide layer after RTA**: **(a) **900°C, 15 s; **(b) **1000°C, 20 s; and **(c) **1050°C, 20 s. The maps of heights are shown on the left-hand side; the same maps with distinguished grain boundaries are shown on the right-hand side. For quantitative parameters see Figure 4.

**Figure 4:**
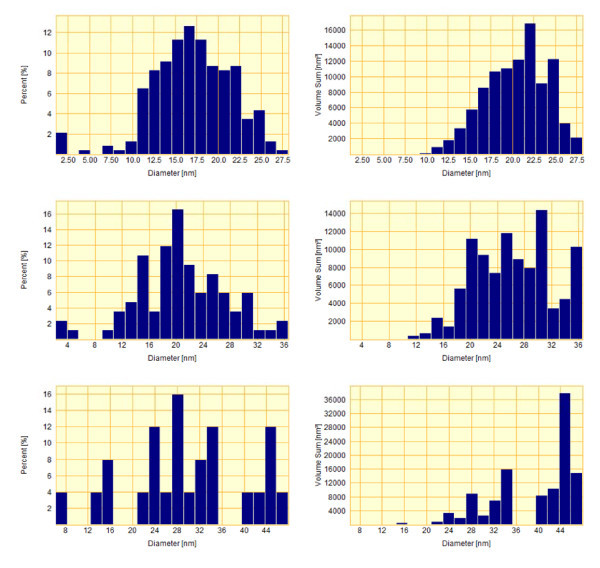
**Histograms of the diameter distributions of gold droplets (left) and the same histograms weighted on droplet volumes (right) (ordinate axis shows the total droplet volume) corresponding to the structures shown in Figure 3**. The density (part of covered surface) of droplets amounts to 912 μm^-2 ^(22.2%), 336 μm^-2 ^(12.7%), and 96 μm^-2 ^(7.1%), respectively for Figure 3 **(a-c)**.

**Figure 5:**
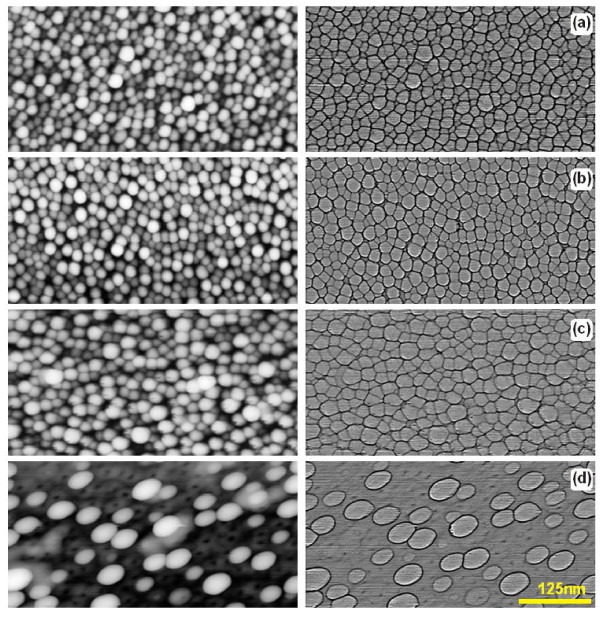
**AFM images of the surfaces of 3-nmthick gold films deposited onto the Wacker-Chemitronic Si substrates with the natural oxide layer after 15 s RTA**: **(a) **400°C; **(b) **700°C; **(c) **950°C; and **(d) **1050°C. The maps of heights are shown on the left hand side; the same maps with distinguished grain boundaries are shown on the right hand side. For quantitative parameters see Figure 6.

**Figure 6:**
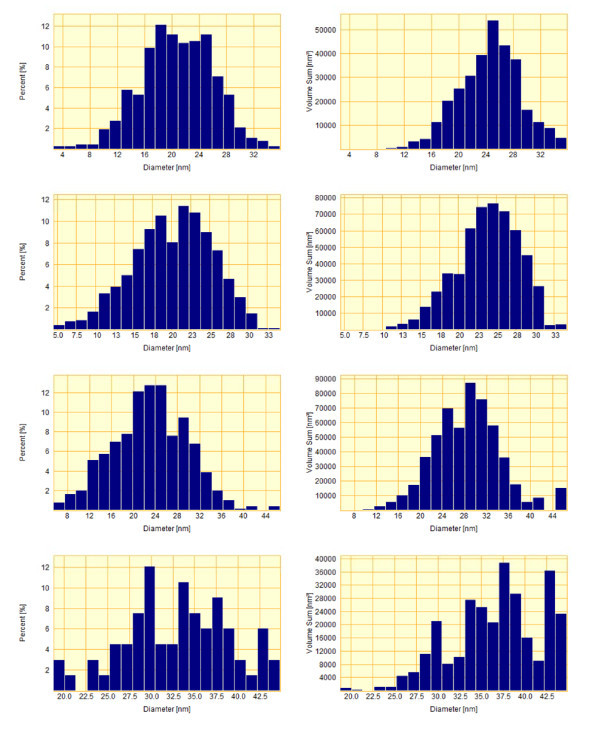
**Histograms of the diameter distributions of gold droplets (left) and the same histograms weighted on droplet volumes (right) (ordinate axis shows the total droplet volume) corresponding to the structures shown in Figure 5**. Droplet density amounts to 2460, 2520, 1940, and 264 μm^-2^, respectively, for **(a-d)**.

Fast anneals at high temperatures of the structures of Si substrates with gold films deposited on them both in furnace and RTA equipment result in the disjoining of gold films and the formation of the arrays of separated gold islands. This process is strongly dependent on the quality of the surface of Si wafers. The results on gold island formation by RTA on the Si wafers procured from Silicon Ltd., and Wacker-Chemitronic are presented in Figures 3, 4, and 5, 6, respectively. It can be seen that the formation of separated gold islands on the Silicon Ltd. wafers (high surface roughness, RMS = 1.83 nm) begins already at 900°C (Figure 3). For the Wacker-Chemitronic Si wafers (RMS = 0.25 nm), the formation of individual gold islands is not observed at any rate up to the temperature of 950°C (see Figure 5). Instead, the individual intergrain boundaries often form joints 120°j for the mentioned temperatures, indicating a steady-state gold film recrystallisation process.

Increase in the RTA temperature resulted in the formation of gold nanoislands on the surface of Si wafers. The size and the density of islands were determined by the annealing temperature (Figures 4 and 6). For both types of substrates used, the typical sizes of nanoislands were in the range of 15-30 nm.

**Figure 7:**
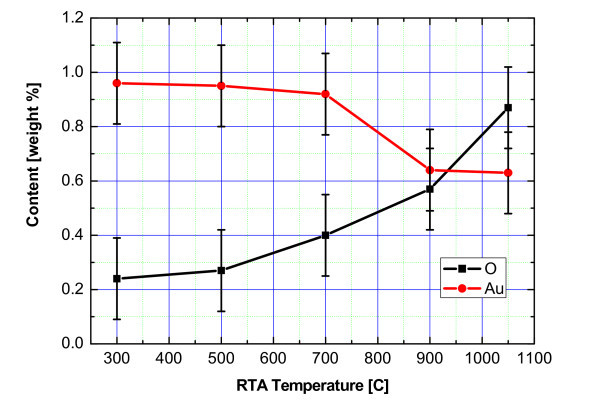
**Dependence of oxygen and gold contents in the subsurface layers of Au/Si structures on the RTA temperature**.

It is worthy to note that the gold evaporation from the Si substrate surface was the accompanying process to the formation of gold island arrays during the high temperature anneals. Figure 7 shows the contents of oxygen and gold in the subsurface layers of Au/Si structures after 15-s RTA treatments at different temperatures for the Wacker-Chemitronic Si wafers. The contents of both gold and oxygen were determined from the results of X-ray energy-dispersive analysis of the scanning electron microscope, Zeiss Evo-50. The integration was carried out over the area of 10 × 10 μm^2 ^with a collection time of 150 s. As can be noted from the data presented, a sharp decrease (by about 1/3) of gold contents in the structures under investigation took place after the threshold temperature of about 800°C. Besides, a nonlinear increase in the oxygen contents in the subsurface layers was observed with the increase of RTA temperature (by a factor of 4 for the temperature range of 300-1050°C). This effect can be due to the presence of oxygen traces in the atmosphere of experimental setup and possible diffusion of oxygen from the substrate bulk to the hetero-boundary in the course of annealing.

**Figure 8:**
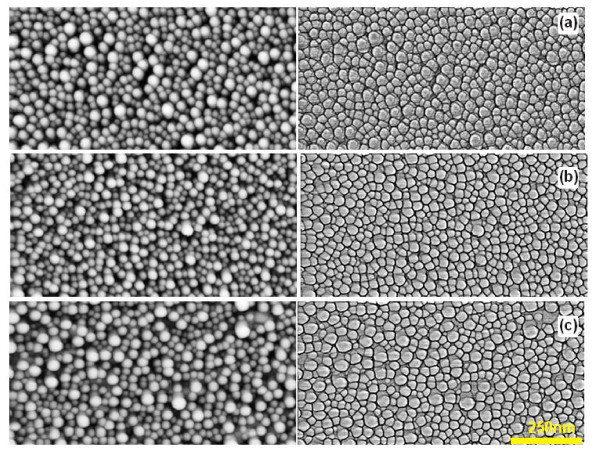
**AFM images of the surface of 5-nm-thick gold film deposited on Wacker-Chemitronic Si substrates with different thicknesses of grown oxide and subjected to 15 s RTA at 950°C**: **(a) **oxide thickness is 1.8 nm; **(b) **oxide thickness is 1.9 nm; **(c) **oxide thickness is 2.0 nm. The maps of heights are shown on the left-hand side; the same maps with distinguished grain boundaries are shown on the right-hand side. For quantitative parameters see Figure 9.

**Figure 9:**
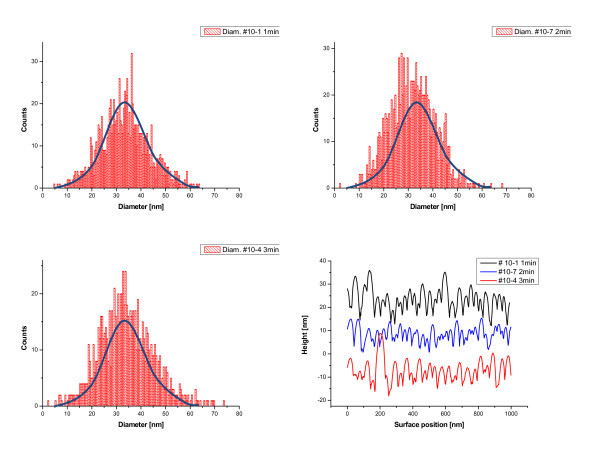
**Histograms of the distributions of the characteristics of structures shown in Figure 8**: gold droplet diameters **(a-c) **and gold film surface profiles **(d)**. For better layout, the distribution **(a) **is superimposed onto the distributions **(b,c)**.

The thickness of oxide layer on the surface of Si wafer had a great effect on the formation of gold nanoisland arrays during the RTA treatments (Figures 8 and 9). One can see that the gold films formed islands more efficiently on the artificial oxide coverage at the RTA temperature of 950°C than on the native oxide coverage. A gradual increase in the oxide thickness promoted the increase in the free space between the grains, in contrast to the closely packed grains on the substrates covered with native oxide. At the same time, the size distribution of grains and its maximum practically did not change with the oxide thickness (Figure 9).

Short furnace anneals of the structures under study under the same conditions as for RTA treatments resulted in the formation of structured gold films with smaller mean grain sizes: 18 nm against 35 nm for the RTA-treated samples. Even at 1024°C, the formation of islands did not take place, although in some films, the regions containing nanopits, from which gold had evaporated, were observed. This difference is unclear at the moment, and needs to be addressed to in more detail in future studies. The authors believe that it can be related to the transition processes during sample heating.

## Conclusions

In this study, the detailed investigations of the peculiarities of the formation of the arrays of gold islands in the course of high temperature anneals of Si wafers with gold films deposited on their surfaces depending on the conditions of wafer surface preparation and annealing regimes are carried out. RTA of Si wafers before the gold deposition was found to smoothen the native oxide layer on their surfaces and stimulate the formation of gold films with bigger crystalline grain structures. RTAs of Au/Si structures at the temperatures 900°C and higher were shown to produce the separate Au droplets on the Si wafer surfaces. Increase of the oxide film thickness on the surface of Si wafers promotes the formation of isolated gold droplets compared to the closely packed droplets formed on the Si surfaces covered with native oxide. Rapid furnace anneals of Au/Si structures were demonstrated not to result in the gold droplet formation but only in the gold film recrystallisation. The results obtained are valuable for the choice of the technological regimes for obtaining the required properties of catalytic gold layers (gold nanodroplet arrays) on the surface of Si substrates for the subsequent growth of Si wire-like crystals.

## Competing interests

The authors declare that they have no competing interests.

## Authors' contributions

AK planned the experiments, took major part in the interpretation of results, participated in the manuscript preparation, AS took part in the interpretation of results and participated in the manuscript preparation, YP and AV made substrate pre-treatments and carried out annealing experiments, OL made AFM investigations, AS made gold film deposition. All authors have read and approved the final manuscript.
